# Health inequities among persons with disabilities: a global scoping review

**DOI:** 10.3389/fpubh.2025.1538519

**Published:** 2025-02-10

**Authors:** Emre Umucu, Andrew A. Vernon, Deyu Pan, Sang Qin, Guillermina Solis, Rebecca Campa, Beatrice Lee

**Affiliations:** ^1^Department of Public Health Sciences, The University of Texas at El Paso, El Paso, TX, United States; ^2^Interdisciplinary Health Sciences, The University of Texas at El Paso, El Paso, TX, United States; ^3^Rehabilitation and Human Services,Penn State Wilkes-Barre, Lehman, PA, United States; ^4^RPSE, University of Wisconsin-Madison, Madison, WI, United States; ^5^College of Nursing, The University of Texas at El Paso, El Paso, TX, United States; ^6^Department of Rehabilitation Sciences, The University of Texas at El Paso, El Paso, TX, United States

**Keywords:** health inequities, disability, health disparities, rehabilitation disparities, scoping review

## Abstract

**Background and objective:**

Approximately 16% of the global population, or 1.3 billion individuals, live with disabilities, facing increased health risks. Despite international and national policies affirming the rights of persons with disabilities, healthcare disparities persist, with studies revealing higher rates of unmet medical needs, avoidable deaths, and dissatisfaction with healthcare services among this population. This scoping review aims to provide a comprehensive overview of health inequities experienced by individuals with disabilities globally.

**Methods:**

A rapid scoping review methodology was employed to systematically search and analyze quantitative evidence on health inequities. Electronic searches were conducted in CINAHL, MEDLINE, and PsycINFO databases, supplemented by manual searches of reference lists. The selection criteria for articles in this study were as follows: (a) publication between 2011 and 2022, (b) written in English, (c) published in a peer-reviewed scholarly journal, and (d) a quantitative comparison of health inequities between persons with and without disabilities.

**Results:**

A total of 363 scholarly works were initially identified, with 51 meeting the inclusion criteria after rigorous screening. In the course of our review, our team identified three overarching themes of health inequity, encompassing (a) access to healthcare and resources, (b) morbidity, mortality, & risk factors, and (c) social determinants of health. These studies collectively reveal disparities in healthcare access, utilization, and outcomes among persons with disabilities, highlighting the urgent need for targeted interventions to address systemic barriers and promote equitable healthcare provision.

**Conclusion:**

This review underscores the challenges faced by individuals with disabilities in accessing quality healthcare and imperative for concerted efforts to advance health equity.

## Introduction

Approximately 16% of the global population, or an estimated 1.3 billion individuals, grapple with various forms of disability, as reported by the World Health Organization (1); moreover, this population confronts more unfavorable health outcomes compared to the general public, with heightened rates of premature mortality and increased morbidity (2–7). For example, among adults, a study revealed that those with cognitive limitations exhibited markedly higher prevalence rates of various health conditions in the United States, including diabetes (19.4% vs. 3.8%), asthma (20.1% vs. 7.8%), arthritis (37.7% vs. 8.3%), cardiac disease (12.7% vs. 3.6%), high cholesterol (29.4% vs. 14.4%), high blood pressure (34.3% vs. 13.8%), and stroke (7.3% vs. 0.4%) compared to their counterparts without cognitive limitations (8). With the growth of this significant population, there has been a corresponding rise in calls to action (9), driving the enhancements in accessibility measures and inclusive practices to address and reduce health inequalities among persons with disabilities.

The United Nations Convention on the Rights of Persons with Disabilities established an international law, affirming the right of persons with disabilities to the highest attainable standards of health, enabling them to make decisions about their bodies and healthcare free from discrimination based on disability (1). Despite the existence of both international and national policies and laws, there are still areas within the healthcare system that do not adhere to these standards. A recent study by Gréaux et al. (9) explored health equity among individuals with disabilities through a global scoping review of barriers and interventions in healthcare services. The authors found that people with disabilities face higher health risks, including increased susceptibility to medical complications and comorbid conditions. Besides, one study examining premature deaths among people with intellectual disabilities (ID) found that individuals with ID (37%) had a higher percentage of avoidable deaths attributable to factors amenable to improvement through quality care, in contrast to the general population (13%) in the UK (10).

Persons with disabilities also experience high dissatisfaction with healthcare services (11). A study reported higher dissatisfaction with healthcare services among those with mild/moderate disabilities (15.88%) and severe/very severe disabilities (19.4%) compared to individuals without disabilities (6.26%) (11). This disparity stems partly from systematic barriers that reflect fundamental issues of alienation and non-inclusiveness, such as the lack of relevant training on disability topics within the healthcare workforce pipeline. Healthcare workers consequently often perpetuate misassumptions and biases in treatment, referrals, or recommendations, which can hinder individuals’ engagement with services (1). Another significant challenge faced by persons with disabilities is limited access to healthcare services. In a study involving 256 healthcare practices, 22% reported their inability to accommodate a fictional patient who has hemiparesis and obesity, uses a wheelchair, and needs assistance in transferring onto the examination table (12).

The human rights approach to health relies on governmental structural interventions, charging countries to safeguard individuals’ healthcare rights, prevent infringements, and fulfill national obligations (1). Despite non-discrimination laws in most countries’ constitutions and legislations, disability is frequently omitted as an explicit basis for discrimination (13). Acknowledging the diverse challenges confronted by persons with disabilities, the United Nations Convention on the Rights of Persons with Disabilities (CRPD), recognizes the elevated risks of discrimination and exclusion, especially for those intersecting with other minoritized identities.^1^ For example, globally, women have a higher prevalence of disability, constituting 19% compared to men’s 12% (14). Notably, women with disabilities undergo screening for exams such as breast and cervical cancer at lower rates than women without disabilities (5). Reasons for these lower screening rates vary, including documented discomfort among service providers in communicating with persons with disabilities (15). Socioeconomic status plays a role in disability and augments perceived stress level and depression in persons with chronic conditions such as those with systemic lupus erythematous (16). On a broader scale, individuals with disabilities encounter additional health inequities that contribute to poorer health outcomes and premature mortality. Notably, adults with ID faced a 3 to 4 times higher risk of death compared to the general population; the mortality rate was even more elevated among those with comorbid epilepsy or Down syndrome (17).

In addition to existing legislation and policy initiatives, crucial improvements are needed to enhance inclusivity, accessibility, and a sense of belonging within the healthcare system. Addressing disparities and inequalities in healthcare for people with disabilities remains an ongoing public and global health concern that warrants further examination. Consequently, this scoping review aims to explore health inequities for individuals with disabilities from a transnational perspective. It marks the initial effort to offer a comprehensive analysis of healthcare service inclusiveness and accessibility for this population. Our goal is to chart this evidence across various elements of healthcare and other contributing factors to its health inequalities, aiming to provide insights that can guide actions taken by governments and other key stakeholders to promote health equity for persons with disabilities.

## Methods

We conducted a rapid scoping review, a systematic and structured approach to rapidly mapping the existing body of literature and identifying knowledge gaps (18, 19). This approach was used to identify quantitative evidence on health inequities experienced by persons with disabilities compared to those without disabilities. The research question guiding this study was: “What are the health inequities for persons with disabilities compared to persons without disabilities?” While we tried to utilize informing elements of the Preferred Reporting Items for Systematic Reviews and Meta-Analyses (PRISMA) protocols (20, 21), the review followed a more flexible and exploratory methodology appropriate for rapidly mapping the existing body of literature.

### Search strategy

An electronic search for studies was conducted in the CINAHL, MEDLINE, and PsycINFO databases. Researchers (18) emphasized the importance of carefully selecting key search terms to thoroughly explore the existing literature. To quantitatively capture information on health inequities between persons with disabilities and those without disabilities, we devised key concepts and search terms. The inclusion criteria stipulated that the terms “disability” OR “disabilities” OR “disabled” had to be present in the title, while search terms such as “health inequity” OR “health disparities” OR “health inequities” OR “health disparity” OR “inequity” were required in the abstract. We recognize that this approach might not capture all relevant studies, as it did not include an exhaustive list of keywords or databases. However, it was tailored to efficiently identify a representative sample of the literature addressing our research question. To supplement the database search, we also conducted a manual review of reference lists in identified articles using Google Scholar to uncover additional sources, including grey literature.

**Figure 1:**
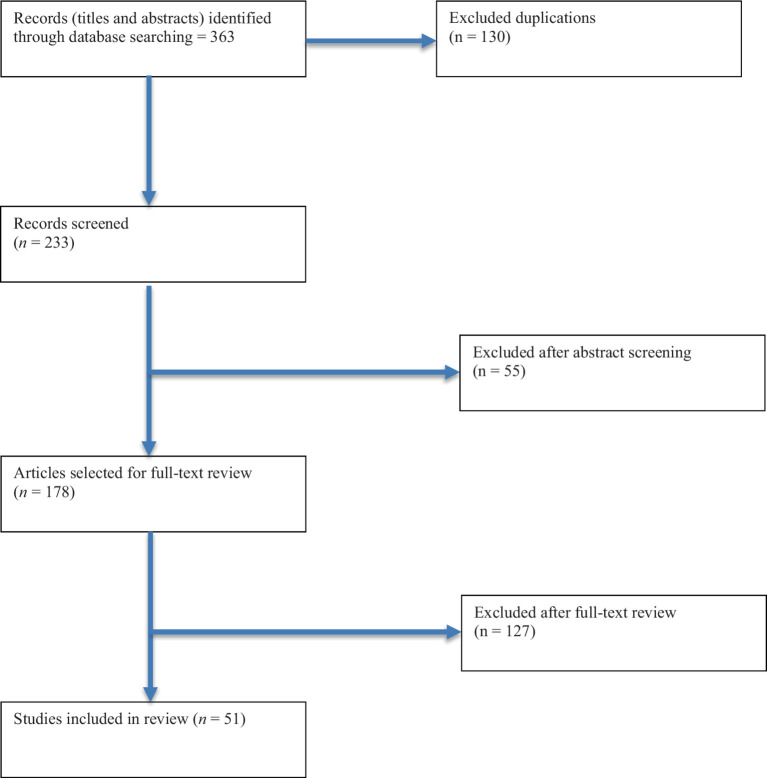
Flowchart of studies.

The selection criteria for articles in this study were as follows: (a) publication between 2011 and 2022, (b) written in English, (c) published in a peer-reviewed scholarly journal, and (d) a quantitative comparison of health inequities between persons with and without disabilities. Following the literature search, a total of 363 scholarly works were identified. An initial screening phase removed 130 manuscripts due to duplication. The remaining 233 articles then underwent a comprehensive review process conducted independently by two co-authors. This multi-stage review process included assessments at the title, abstract, and full manuscript levels to ensure that only studies meeting the inclusion criteria were retained. During the title and abstract review, the two reviewers independently screened articles to identify studies with a focus on quantitative comparisons of health inequities between persons with and without disabilities. At this stage, 55 scholarly works were excluded because they did not meet the inclusion criteria, such as failing to compare groups with and without disabilities. In the next phase, full-text reviews were conducted on the remaining articles to assess their relevance and eligibility. The two reviewers collaborated closely to resolve discrepancies and ensure consistent application of the inclusion criteria. During this process, an additional 127 manuscripts were excluded, primarily due to being inaccessible or irrelevant to the study objectives. Ultimately, 51 scholarly works were identified for inclusion in the review. These studies represent the body of evidence used to explore health inequities between people with and without disabilities. Figure 1 provides a detailed flow chart outlining the screening and selection process.

## Results

The World Health Organization (WHO) defines “health inequities” as systematic differences in the health status of various population groups (1). Irrespective of race/ethnicity, culture, socioeconomic status, and nationality, individuals with disabilities encounter disparities in terms of health outcomes and health risks worldwide. In the course of our review, our team identified three overarching themes of health inequity, encompassing (a) access to healthcare and resources, (b) morbidity, mortality, & risk factors, and (c) social determinants of health. Aligning with this conceptualization, we synthesized quantitative evidence on prevailing health inequities for individuals with disabilities in comparison to those without disabilities in the global context (Table 1).

**Table 1:** Study characteristics and findings.

Article #	Citation	Year	Location	Sample	Study type	Notes
1	Heslop et al. (46)	2021	UK	Persons with ID	Quantitative	Explored the circumstances leading to death from COVID-19 in persons with ID and found that persons with ID had different COVID-19 symptoms and age at death compared to the general population. For example, none of the persons with ID who died from COVID-19 reported an altered sense of smell or taste, meaning that it was difficult for healthcare providers to identify this symptom.
2	Feldner et al. (34)	2022	U.S.	Occupational and physical therapy assistants	Quantitative	Explored explicit and implicit disability attitudes in occupational and physical therapy assistants and reported that although the majority of occupational and physical therapy assistants reported no explicit preference for persons with disabilities or persons without disabilities, the majority occupational and physical therapy assistants were aversive ableists.
3	Desroches et al. (33)	2022	Australia	Nurses	Quantitative	Examined nurses’ attitudes and emotions toward caring for persons with ID and reported that nurses’ attitudes toward persons with ID were significantly less positive compared to persons without ID.
4	Fisher et al. (29)	2020	U.S.	Nurses	Review	Aimed to investigate the knowledge gaps among healthcare professionals in meeting the needs of individuals with IDD throughout their lives and identify the factors contributing to these gaps and reported that insufficient knowledge and understanding among nurses about individuals with IDD exacerbate health disparities in healthcare and services.
5	Woodard et al. (32)	2012	U.S.	Medical students	Program Evaluation	Aimed to address the lack of medical training in caring for persons with disabilities and improve their health outcomes and reported that their training module integrated into the primary care at USF improved medical students’ knowledge, attitudes, and comfort in caring for persons with disabilities.
6	Edwards et al. (28)	2022	U.S.	Nursing students	Quantitative	Examined the impact of clinical contact with persons with disabilities on nursing students’ knowledge, skills, and attitudes and reported that clinical exposure positively impacted nursing students’ attitudes and skills in caring for persons with disabilities.
7	Smith et al. (30)	2011	U.S.	Pharmacy students	Review/Perspective	Aimed to define disability as a culture and explain how this concept can be integrated into cultural competency education and reported that incorporating disability education into pharmacy curricula to enhance healthcare providers’ knowledge and skills in caring for individuals with disabilities.
8	Wilkinson et al. (31)	2012	U.S.	Family physicians	Qualitative	Investigated and identified areas of discomfort or need among practicing physicians to more clearly define the key elements that should be incorporated into medical school or residency curricula and reported that family physicians caring for persons with ID lack experience and confidence in providing care for this population.
9	Heslop et al. (10)	2014	UK	Persons with ID	Population-based study	Investigated the factors contributing to premature deaths in persons with ID in England and reported that persons with ID experience significantly higher rates of premature death compared to general population due to factors like poor care planning.
10	Mutwali et al. (22)	2019	South Africa	Persons with disabilities	Quantitative	Compared disparities in physical access and healthcare utilization between persons with and without disabilities and reported that more than half (52%) of the households with a member with disability were found to have poor physical access to healthcare compared to only 47% households without a disabled member.
11	Lee et al. (23)	2012	U.S.	Older adults with disabilities	Quantitative	Compared delays and disparities in seeing a doctor among older persons with and without disabilities and reported that (a) significantly more older persons with disabilities (5.84%) experienced medical care delay due to cost than their counterparts and (b) older adults with disabilities had significantly higher rates of diseases such as diabetes (24.23% vs. 15.67), asthma (16.08% vs. 7.94%) compared to older persons without disabilities.
12	Trani et al. (11)	2011	Sierra Leone	Persons with disabilities	Quantitative	Compared health status and access to health care services between persons with and without disabilities and found that (a) persons with severe disabilities had less access to public health care services compared to persons without disabilities and (b) women with disabilities were as likely to report access to maternal health care services as did women without disabilities.
13	Mahmoudi et al. (4)	2015	U.S.	Persons with physical disabilities	Quantitative	Compared disparities in access to healthcare in persons with and without physical disabilities and reported that compared to persons without physical disabilities, those with physical disabilities had 1.75 times greater adjusted odds of unmet medical care, 1.57 times for dental care, and 1.85 times lack of medication care.
14	Walker et al. (25)	2016	U.S.	Parents and children with disabilities	Qualitative	Examined health disparities in the context of the barriers and facilitators to accessing health and support services among urban and rural parents of children with disabilities and found that (a) parents living in rural areas were required to travel long distances to receive necessary and recommended care services for their child with disability and (b) parents living in urban areas experienced high care costs although more services were available form them compared to their rural counterparts.
15	Okoro et al. (50)	2014	U.S.	Person with and without disabilities	Quantitative	Examined severity of psychological distress in persons with and without disabilities and reported that (a) persons with disabilities had a higher prevalence of mild to moderate and serious psychological distress compared to persons without disabilities, (b) persons with disabilities who are not working had significantly higher prevalence estimates of moderate and serious psychological distress, and (c) persons with disabilities who had either moderate or serious psychological distress were significantly more likely than those with no distress to be physically inactive and to be current smokers.
16	Ko et al. (3)	2011	Korea	Persons with disabilities	Quantitative	Compared disparities in health risk behaviors, preventive healthcare utilization, and chronic conditions between persons with and without disabilities and reported that significantly more people with disabilities were physical inactive (8.45% vs. 2.26%), had osteoporosis (17.28% vs. 9.88%), were underweight (7.55% vs. 4.12), had suicidal thoughts (35.21% vs. 19.16%), and had lower quality of life (63.12% vs. 70.39%) compared to those without disabilities
17	Moodley et al. (6)	2015	South Africa	Persons with disabilities	Quantitative	Compared inequities in health outcomes and access to health care between persons with and without disabilities and reported that (a) more persons with disabilities (6%) diagnosed with tuberculosis than persons without disabilities (3%) and (b) the prevalence rates of diabetes (8% vs. 2%), stroke (3% vs. 1%), asthma (6% vs. 3%), and heart problems (8% vs. 2%) were significantly higher in persons with disabilities compared to those without disabilities.
18	Young-Southward et al. (57)	2017	UK	Persons without ID	Quantitative	Examined physical and mental health of young persons with and without ID and reported that persons with ID were 9.6 to 125 times more likely to have poor health on the seven outcomes investigated than were those without intellectual disabilities (i.e., general health, mental health, physical disabilities, hearing impairment, visual impairment, long-term illness and day-to-day activity limitations).
19	Ross et al. (47)	2020	U.S.	Children and adolescents with disabilities	Quantitative	Estimated the population level physical activity and sports participation in children and adolescents with disabilities and reported that (a) the sports participation rates for children (33.74%) and adolescents with disabilities (40.76%) were significantly lower than the participation rate for children (49.89%) and adolescents without disabilities (59.94%) and (b) children with disabilities (23.82%) were significantly less physically active than children without disabilities (28.45%).
20	Okoro et al. (48)	2020	U.S.	Persons with disabilities	Quantitative	Compared short sleep duration among persons with and without disabilities and reported that persons with any disability (43.8%) had significantly higher prevalence of short sleep duration compared to those without disabilities (31.6%), meaning that persons with disabilities sleep shorter than recommended sleep time for healthy adults.
21	Barnhart et al. (52)	2020	U.S.	Persons with disabilities	Mixed methods	Explored smoking behaviors and health status and reported that persons with disabilities disproportionally use tobacco and experience negative health consequences associated with tobacco use, and accessible health promotion smoking cessation interventions may help improve health and achieve health equity for persons with disabilities.
22	Elia et al. (2)	2021	U.S.	Persons with disabilities	Quantitative	Estimated the prevalence of nephrolithiasis in persons with disabilities and found that the prevalence of nephrolithiasis in persons with disabilities is 16.1% in comparison to 9.2% in persons without disabilities.
23	Reichard et al. (24)	2011	U.S.	Persons with physical disabilities or cognitive limitations	Quantitative	Explored health disparities in persons with physical disabilities or cognitive limitations compared to those with no disabilities and reported that compared to persons without disabilities (a) persons with physical disabilities or cognitive limitations were found to have higher prevalence rates for arthritism asthma, cardiovascular disease, diabetes, high blood pressure, high cholesterol, and stroke and (b) the disability groups were also significantly less likely to receive preventative care.
24	Heinrichs et al. (35)	2018	Canada	Children with DD	Quantitative	Explored health and health care utilization in children in care with and without DD and reported that children with DD had significantly more likely to have a history of mood and anxiety disorders, respiratory illnesses, diabetes, hospital-based dental care, and injury-related hospitalizations, and made more ambulatory physician visits compared to children without DD.
25	Shin et al. (54)	2020	South Korea	Persons with disabilities	Quantitative	Examined disparities in the participation rate of colorectal cancer screening and found that (a) persons with severe disabilities had a lower levels of colorectal cancer screening participation rate compared to persons without disabilities and (b) people with autism, renal failure, brain injury, ostomy, and ID had the lowest participation rates.
26	Jarrett et al. (43)	2013	U.S.	College students with disabilities	Quantitative	Compared cigarette smoking between college students with and without disabilities and reported that (a) college students with disabilities (23.1%) had higher smoking prevalence compared to those without disabilities (15%) and (b) college students with psychiatric disabilities had the highest smoking rate (29.9%), followed by those with learning disabilities (23.7%), sensory disabilities (19.8%), and physical disabilities (16.4%).
27	Austin et al. (58)	2016	U.S.	Persons with disabilities	Quantitative	Explored adverse childhood experiences in persons with disabilities and found that compared to those without disabilities (a) persons with disabilities reported higher levels of adverse childhood experiences (b) among those with high adverse childhood experiences exposure, persons with disabilities were found to be at increased risk for certain health risks (e.g., smoking) and perceived poor health (e.g., mental health).
28	Reichard and Stolzle (8)	2011	U.S.	Persons with cognitive limitations	Quantitative	Explored diabetes in persons with cognitive limitations compared to persons without cognitive disabilities and found that compared to those without cognitive disabilities (a) persons with cognitive limitations had significantly higher prevalence rate of diabetes, asthma, arthritis, cardiac disease, high cholesterol, high blood pressure, and stroke.
29	Shooshtari et al. (36)	2017	Canada	Persons with DD	Quantitative	Compared health status, health trajectories and use of health and social services between children with and without DD and found that children with DD were significantly more likely to die before the age of 17 and have a history of respiratory illness, diabetes, and illness-related hospitalizations compared to children without disabilities.
30	Reichard et al. (42)	2019	U.S.	Persons with IDD	Quantitative	Examined characteristics of Medicare beneficiaries with IDD and found that persons with IDD who utilize Medicare had higher prevalence of COPD, congestive health failure, diabetes, hypertension, and obesity.
31	Emerson et al. (56)	2019	UK	Persons with ID	Quantitative	Examined the risk of exposure to air pollution in children with and without ID and found that children with ID had significantly higher levels of exposure to outdoor air pollution compared to children without ID, contributing to the health inequities.
32	Berg et al. (59)	2019	U.S.	Children with DD	Quantitative	Examined the prevalence of adverse experiences among children with DD and reported that children with DD were more likely to experience higher levels of adverse family experiences compared to children without DD, contributing to the health inequities.
33	McMahon et al. (41)	2021	UK	Persons with ID	Quantitative	Compared the prevalence of health problems in persons with and without ID and found that persons with ID were more likely than those without ID to have viral or infective diseases, mental health illnesses and behavioral problems, neurological disorders, diseases of the genitourinary system and malformations or genetic problems and less likely to have cancer, and musculoskeletal diseases.
34	Zhang et al. (60)	2018	China	Persons with disabilities	Quantitative	Examined the gender disparities in the relationship between sociodemographic variables and non-communicable diseases and reported that women with disabilities were about 11.6% points more likely to suffer from high blood lipids and less likely to develop high blood pressure, high blood glucose, and being overweight compared to men.
35	Morin et al. (40)	2012	Canada	Persons with ID	Quantitative	Compared the prevalence of chronic diseases in persons with and without ID and found that those with ID had higher rates of heart disease and thyroid disorder and less likely to have arthritis, migraines, back or spinal pain, and food allergies compared to those without ID.
36	Froehlich-Grobe et al. (49)	2013	U.S.	Persons with disabilities	Quantitative	Examined disparities in obesity and related conditions in persons with disabilities and reported that (a) the prevalence rates of obesity and extreme obesity were significantly higher in persons with disabilities compared to those without disabilities and (b) persons with disabilities were significantly more likely to report being told they had high cholesterol, hypertension, or diabetes.
37	Mesfin (26)	2021	Ethiopia	Adolescents with disabilities	Quantitative	Compared disparities in sexual and reproductive health service utilization between adolescents with and without disabilities and reported that adolescents with disabilities (32.2%) utilized sexual and reproductive health services significantly less than adolescents without disabilities (51.7%).
38	Carter et al. (27)	2021	Mixed	Persons with ID	Scoping Review	Examined the sexual and reproductive health and rights of young persons with ID and found that although there are positive developments of sexual and reproductive agency for persons with ID, there are still several barriers including negative attitudes and infantilization of young persons with ID, leading to health inequities.
39	Dembo et al. (51)	2018	U.S.	People with disabilities	Quantitative	Compare the psychological consequences of violence between persons with and without disabilities and found that women with disabilities were more likely than men and women without disabilities to report severe distress from violence, causing significant negative psychological consequences.
40	Brennand et al. (53)	2022	Canada	Persons with disabilities	Quantitative	Explored the relationship between disability and sexually transmitted diseases (STIs) and found that persons with disabilities were more likely to report having being diagnosed with STIs compared to those without disabilities.
41	Mitra et al. (38)	2016	U.S.	Women with disabilities	Quantitative	Compared disparities in adverse preconception risk factors between women with and without disabilities and reported that (a) the prevalence rates of smoking (30.5% vs. 14.5%) and diabetes (12.5% vs. 5.6%) were significantly higher in women with disabilities compared to women without disabilities, indicating that women with disabilities at reproductive age were more vulnerable to risk factors associated with adverse pregnancy outcomes than women without disabilities.
42	Brown et al. (39)	2016	Canada	Women with IDD	Quantitative	Compared pregnancy in women with and without IDD and found that compared to women without IDD (a) general fertility rate was lower in women with IDD and (b) women with IDD were younger and experienced more poverty, epilepsy, obesity, and mental health issues.
43	Kim et al. (70)	2013	U.S.	Women with disabilities	Quantitative	Compared health disparities in childrearing women with and without disabilities and found that compared to those without disabilities (a) women with disabilities were less likely to have a partner or spouse, report lower education levels and income and are older and (b) women with disabilities had poorer health-related quality of life, higher prevalence of chronic health conditions (i.e., arthritis, diabetes, cardiovascular disease, asthma, high blood pressure and cholesterol, and obesity), higher prevalence of adverse health behaviors (i.e., smoking, lack of exercise), more financial barriers to health care and lower levels of social and emotional support.
44	Slayter (61)	2016	U.S.	Women with IDD	Quantitative	Compared disparities in substance abuse treatment utilization between women with IDD and men and women without disabilities and found that women with IDD were less likely to utilize substance abuse treatment compared to men and women without disabilities.
45	Bussiere et al. (55)	2014	France	Women with disabilities	Quantitative	Examined the effects of obesity and mobility in access to breast and cervical cancer screening in community dwelling women and found that women with higher BMI or disability score had lower likelihood getting Pap test and mammogram use.
46	Brown et al. (45)	2021	Canada	Women with disabilities	Quantitative	Examined the association between preexisting disability and severe maternal morbidity or mortality and found that “compared with women without a disability, the adjusted relative risk of severe maternal morbidity or death was 29% higher among women with a physical disability, 14% higher among women with a sensory disability, 57% higher among women with an intellectual/developmental disability, and 74% higher among women with 2 or more disabilities,”
47	Warner et al. (63)	2011	U.S.	Persons with disabilities	Quantitative	Examined how race/ethnicity and gender define age-trajectories of disability in White, Black, and Mexican American Men and Women and reported that (a) Black and Hispanic women had the highest disability levels and (b) women from all racial and ethnic groups had higher levels of functional limitations compared to all men.
48	Magana et al. (65)	2016	U.S.	Persons with IDD	Quantitative	Explored racial and ethnic disparities in persons with IDD and reported that Latino and Black persons with IDD were found to have worse health outcomes compared to White persons with IDD.
49	Siordia et al. (64)	2017	U.S.	Persons with disabilities	Quantitative	Explored prevalence and risk for negative disability outcomes between American Indians-Alaskan Natives (AIANs) and other race/ethnic groups and reported that AIANs had higher risk for disability than non-Hispanic White people, non-Hispanic Asians, and Hispanics.
50	Whitson et al. (62)	2011	U.S.	Persons with disabilities	Quantitative	Compared Black-White disparity in disability and reported that Blacks were more likely to be obese and have diabetes, and less likely to report vision problems, fractures, and heart attacks compares to Whites.
51	Emerson et al. (66)	2019	UK	Persons with and without disabilities	Quantitative	Compared perceived discrimination in working-age persons with and without disabilities and reported that persons with disabilities were over three times more likely than their counterparts to be exposed to discrimination.

### Access to healthcare and resources

Persons with disabilities may require more frequent primary care screenings to enhance their health and rehabilitation outcomes. However, they often encounter challenges such as costs and transportation when trying to access quality healthcare and resources, leading to health inequities. The disparities in access to healthcare services and resources for individuals with disabilities represent a significant global social justice issue. Our review extensively documented substantial and systematic evidence illustrating persons with disabilities consistently face challenges and disparities in accessing healthcare and resources compared to their counterparts without disabilities.

#### Unmet healthcare access needs and poor-quality care

In the U.S., researchers conducted a study to assess healthcare access among individuals with and without physical disabilities (4). The findings revealed that individuals with physical disabilities faced higher odds of encountering unmet medical care (1.75 times), dental care (1.57 times), and not receiving medications when needed (1.85 times) compared to those without physical disabilities. Similarly, another study by researchers focused on investigating premature deaths in individuals with ID in the UK with the goal of identifying contributory factors to avoidable and premature deaths in this population (10). The study disclosed that individuals with ID experienced a significantly higher percentage of avoidable deaths (37%) from causes amenable to the improvement of care compared to the general population (13%) in the U.K. These avoidable premature deaths within a subset of individuals with ID can be attributed to a few factors: challenges in advanced care planning, poor adherence to the Mental Capacity Act legislation related to assessment of a person’s capacity to make healthcare decisions, living in inappropriate accommodation, failing to adapt care as needs changed, and caregivers not feeling being listened to.

In Sierra Leone, 83.58 to 92.86% of persons with varied severity of disabilities reported having healthcare access, in contrast to non-disabled individuals, whose access was reported at 97.71% (11). The researchers further highlighted that a higher percentage of persons with mild/moderate disabilities (15.88%) and those with severe/very severe disabilities (19.4%) expressed dissatisfaction with healthcare services compared to persons without disabilities (6.26%) (11). Meanwhile, researchers in South Africa documented that over 52% of households with members experiencing disabilities faced challenges related to poorer physical access to healthcare (22). In comparison, 47% of households without members with disabilities encountered similar difficulties.

#### Financial and geographical barriers

Discussions on barriers to accessing healthcare for individuals with disabilities often revolve around economic challenges and high medical costs. One study documented that a significantly greater proportion of older adults with disabilities (5.84%) experienced delays in seeing a doctor due to costs compared to older adults without disabilities (2.57%) in the U.S (23). A different U.S. study found that individuals with cognitive disabilities incurred significantly higher healthcare costs ($8,099) compared to those without disabilities ($2,068) (8). The researchers further noted that individuals with both cognitive limitations and diabetes experienced 3.7 times higher healthcare expenditures ($16,457) compared to individuals with diabetes but without cognitive limitations ($4,490) (8). In yet another U.S. study, researchers reported that individuals with cognitive disabilities (4.8 times) and those with physical disabilities (4.3 times) had higher medical expenditures compared to individuals without disabilities (24).

Geographical location, such as rural versus urban settings, has also been scrutinized as a barrier to accessing healthcare for individuals with disabilities (23, 25). Researchers reported that a significantly higher percentage of individuals with disabilities (22.36%) resided in rural areas compared to those without disabilities (20.95%) in the U.S (23).

#### Access to sexual and reproductive healthcare

Individuals with disabilities may encounter more obstacles in accessing sexual and reproductive healthcare services and resources in comparison to those without disabilities. In Ethiopia, researchers discovered that significantly fewer adolescents with disabilities (32.2%) utilized sexual and reproductive health services compared to their counterparts without disabilities (51.7%) (26). Furthermore, their study highlighted that, in contrast to adolescents without disabilities (69.7%), a greater proportion of adolescents with disabilities (72%) exhibited poor knowledge of sexual and reproductive health (26).

Another study documented that young adults with ID accessed sexual knowledge through different channels, such as relying on family, compared to young adults without disabilities who often used platforms like social media (27). However, young adults with ID face significant barriers, including negative attitudes and infantilization. For instance, compared to adolescents without disabilities (41.7%), a lower proportion of adolescents with disabilities (18.5%) had discussed sexual and reproductive health issues with healthcare workers in the past 12 months in Ethiopia (26). This is crucial, as adolescents with disabilities who had discussed such issues with healthcare workers were 2.3 times more likely to use sexual and reproductive health services than those with disabilities not discussed (26).

#### Providers’ competency and attitudes

Despite limited quantitative data, numerous researchers have consistently highlighted that healthcare providers, including medical school students, nurses, physicians, pharmacy students, and nursing students, may encounter challenges in delivering appropriate and quality healthcare services to individuals with disabilities (28–32). This is attributed to a lack of training, experience, and knowledge concerning disability-specific issues. The absence of adequate training in disability-related matters can leave healthcare providers ill-equipped to offer quality care to individuals with disabilities, thereby contributing to health inequities (30–32).

Furthermore, healthcare providers’ attitudes have been identified as an additional barrier to accessing quality healthcare. A study reported that nurses in Australia exhibited significantly fewer positive attitudes toward individuals with intellectual disabilities than toward those without intellectual disabilities (33). Another study demonstrated that a majority of occupational and physical therapist assistants held aversive ableist attitudes, resulting in a bias against persons with disabilities in their professional interactions in the United States (34). Notably, the research found that 80.1% of occupational and physical therapist assistants preferred working with persons without disabilities, while only a small percentage (7.6%) expressed a preference for working with persons with disabilities (34).

### Morbidity, mortality, and risk factors

Our review indicates that persons with disabilities are more prone to having both comorbid physical and mental health conditions compared to their counterparts without disabilities. Moreover, there is an elevated risk of premature deaths among this population. We additionally identified several risk factors contributing to adverse health outcomes in three board categories: lifestyle behaviors, psychosocial factors, and structural issues.

#### Comorbid physical health conditions

In contrast to their non-disabled peers, people with disabilities are more likely to develop comorbid physical conditions. A study conducted in Canada found that a significantly higher percentage of children with developmental disabilities (DD) had diabetes (1.40% vs. 0.66%), injury-related hospitalization (1.90% vs. 0.83%), and hospital-based dental care (7.51% vs. 4.13%) compared to their counterparts without DD (35). Another research study in Canada revealed that children with DD were approximately three times more likely to have diabetes compared to children without disabilities (36).

Among adults, a study (8) found that those with cognitive limitations had significantly higher prevalence rates of various health conditions in the U.S., including diabetes (19.4% vs. 3.8%), asthma (20.1% vs. 7.8%), arthritis (37.7% vs. 8.3%), cardiac disease (12.7% vs. 3.6%), high cholesterol (29.4% vs. 14.4%), high blood pressure (34.3% vs. 13.8%), and stroke (7.3% vs. 0.4%), compared to adults without cognitive limitations. Another study in the U.S. revealed that individuals with disabilities (16.1%) had a significantly higher prevalence rate of nephrolithiasis than individuals without disabilities (9.2%) (2). Furthermore, it was reported that individuals with physical disabilities, compared to those without physical disabilities, exhibited a higher incidence of chronic physical conditions, such as diabetes (19% vs. 5%), asthma (19% vs. 8%), high blood pressure (50% vs. 22%), any heart problems (23% vs. 7%), joint problems (75% vs. 27%), and arthritis (58% vs. 14%) (4).

Among women, Kim and colleagues reported significantly higher percentages of childrearing women with disabilities experiencing poorer general health (31.88% vs. 6.06%) and poorer physical health (32.46% vs. 4.43%) compared to childrearing women without disabilities in the U.S (37). The study also highlighted elevated prevalence rates of asthma (31.68% vs. 13.84%), arthritis (44.14% vs. 10.63%), obesity (36.33% vs. 20.06%), diabetes (7.58% vs. 2.14%), high blood pressure (21.78% vs. 8.63%), high cholesterol (31.22% vs. 20.26%), heart attack (1.99% vs. 0.37%), angina (2.16% vs. 0.45%), and stroke (2.05% vs. 0.47%) among childrearing women with disabilities. In another study it was reported that significantly more women with disabilities experienced mental distress (34.7% vs. 9.4%), obesity (37.5% vs. 22.8%), and asthma (29.1% vs. 13.5%) compared to women without disabilities in the U.S (38). Furthermore, researchers found that women with intellectual and developmental disabilities (IDD) in Canada had significantly lower general fertility rates (20.3 per 1,000) compared to women without IDD (43.4 per 1,000) (39).

Specifically for individuals with intellectual disabilities (ID), a comparative study on the prevalence of chronic conditions in persons with and without ID in Canada revealed significantly higher rates of heart disease (7.2% vs. 5.1%) and hypo- or hyperthyroidism (11.2% vs. 6.7%) among persons with ID compared to the general population without ID (40). Similarly, another study highlighted that individuals with ID were more likely to have viral or infective diseases (7.8% vs. 2.5%); neurological disorders (30% vs. 4.7%); blood diseases (7.4% vs. 3.1%); endocrine, nutritional, and metabolic conditions (30.9% vs. 19.9%); eye diseases (18.9% vs. 8.8%); respiratory system diseases (19.4% vs. 13.4%); digestive system diseases (34.6% vs. 15.2%); skin diseases (30.9% vs. 14.5%); and diseases of the genitourinary system and malformations or genetic problems (30% vs. 8.3%) compared to the general population without ID (41).

Among older adults with Medicare, individuals with intellectual and developmental disabilities (IDD) and related conditions exhibited considerably higher prevalence rates for chronic obstructive pulmonary disease (COPD) (34.9% vs. 14.4%), congestive heart failure (41% vs. 14.3%), diabetes (47% vs. 27.9%), hypertension (84.7% vs. 70.1%), and obesity (17.3% vs. 10%) compared to beneficiaries with no disabilities (42). Similarly, researchers reported that older individuals with disabilities, in comparison to their counterparts without disabilities, had a higher incidence of chronic physical conditions in the U.S., including diabetes (24.23% vs. 15.67%), asthma (16.08% vs. 7.94%), heart attack (19.79% vs. 10.56%), coronary heart disease (21.49% vs. 10.90%), and stroke (13.58% vs. 5.37%) (23).

#### Comorbid mental health conditions

Similar to comorbid physical health conditions, individuals with disabilities are more likely to experience comorbid mental health conditions compared to those without disabilities. In Canada, researchers found that a significantly higher percentage of children with DD (19.76%) had mood and anxiety disorders compared to those without DD (11.21%) (35). In South Korea, a study reported that individuals with disabilities (35.21%) had a higher proportion of suicidal ideation compared to individuals without disabilities (19.16%). The same researchers also found that a higher proportion of individuals with disabilities (22.54%) reported depression compared to those without disabilities (15.28%) (3). Other researchers noted that college students with disabilities in the U.S. experience significantly higher prevalences of stress (63.1% vs. 46.6%, respectively) and depression (27.9% vs. 5.7%, respectively) compared to college students without disabilities (43). Similarly persons with disabilities in the United Kingdom were among the largest group of people affected by loneliness and often lead to depression and self-isolation with poor health outcomes (44).

Furthermore, a study revealed that individuals with intellectual disabilities (ID) (52.5%) were more likely to have mental illnesses and behavioral problems compared to the general population without ID (15%) (41). Kim and colleagues reported that childrearing women with disabilities had more frequent mental distress (28.94% vs. 8.83%) and lacked social and emotional support (9.23% vs. 4.38%) compared to childrearing women without disabilities in the U.S. (37). In another Canadian study, Brown and colleagues reported that women with disabilities (17.3 to 37.6%) were more likely to have mental illness compared to those without disabilities (12.6%) (45). More specifically, women with IDD (37.6%) had the highest prevalence of mental illness compared to those with sensory disability (17.3%), physical disability only (19.7%), and multiple disabilities (26.8%).

#### Mortality

Given the poor health outcomes and comorbidities, premature deaths are more prevalent among individuals with disabilities compared to those without disabilities. In Canada, a study revealed that children with developmental disabilities (DD) were eight times more likely to die before the age of 17 compared to those without disabilities (36). In the context of maternal health, compared to women without disabilities (1.7%), women with a physical (2.4%), sensory (2.1%), intellectual and developmental disability (IDD) (3.0%), and two or more disabilities (3.5%) had a significantly higher risk of severe maternal morbidity or death (45). Among older adults, researchers revealed that mortality within 30 days of hospitalization among Medicare beneficiaries with IDD who were 65 or older (1.7%) was about twice as high compared to those beneficiaries without disabilities (0.9%) in the U.S. (42). Finally, mortality rates during COVID-19 for individuals with ID were examined, and it was found that only 4% of individuals with ID who died from COVID-19 were 85 years or older, whereas 47% of deaths from COVID-19 were individuals without ID who were 85 or older (46).

#### Risk factors of health outcomes

Several risk factors contributing to adverse health outcomes have been identified in individuals with disabilities, encompassing physical inactivity, sleep issues, obesity, psychological distress, smoking, alcohol and drug use, sexually transmitted infections, and a lack of health screening.

##### Physical inactivity

A recent U.S. study revealed that in comparison to children (49.89%) and adolescents (59.94%) without disabilities, a lower percentage of children (40.76%) and adolescents (40.76%) with disabilities participated in sports teams and lessons (47). Additionally, researchers reported that the rate of physical activity participation among children with disabilities (49.89%) was significantly lower than that among children without disabilities (28.45%) (47). Another study in South Korea found that individuals with disabilities (8.45%) were significantly more likely to be physically inactive compared to those without disabilities (2.26%) (3). Kim and colleagues reported that significantly more childrearing women with disabilities were physically inactive (25.34% vs. 16.52%) compared to childrearing women without disabilities in the U.S (37). Finally, another study revealed that women with disabilities were significantly less likely to have exercised in the past month (67.1% vs. 79.8%) compared to women without disabilities (38).

##### Sleep issues in relation to obesity

A study reported that adults with disabilities (43.8%) had a significantly higher prevalence of short sleep duration compared to adults without disabilities (31.6%) in the U.S (48). BMI was assessed as a risk factor, and researchers found that a higher percentage of adults with disabilities (36.8%) had a BMI of 30 or above compared to adults without disabilities (24.8%) (48). Furthermore, individuals with disabilities were observed to have a significantly greater waist circumference (100.5 cm vs. 95.8 cm) and a higher percentage of body fat (35.7% fat vs. 33.4% fat) than those without disabilities (49).

##### Psychological distress

Psychological distress is more prevalent in persons with disabilities compared to persons without disabilities. One research study showed that significantly more adults with disabilities reported mild-to-moderate (16.1% vs. 6.7%) and severe (9.5% vs. 2.1%) psychological distress compared to adults without disabilities (50). As expected, among those with disabilities, significantly higher rates of adults with either mild-to-moderate (46.1% vs. 33.6%; 35.1% vs. 19.1%, respectively) or severe (52.2% vs. 33.6%; 43.9% vs. 19.1%, respectively) psychological distress were physically inactive and current smokers compared to those with no psychological distress. Another research study also revealed that significantly more men (37.2%) and women (57.5%) with disabilities experienced severe distress compared to men (16.6%) and women (35.1%) without disabilities (51).

##### Smoking

Persons with disabilities disproportionately use tobacco products and experience associated negative health outcomes (52). Researchers have also examined smoking behaviors in persons with disabilities (48). One study revealed that more adults with disabilities were current (24.5%) or former (30.4%) smokers compared to adults without disabilities (13.6 and 22.6%, respectively) in the U.S. Another research study found that smoking prevalence was significantly higher among college students with disabilities (23.1%) compared to those without disabilities (15%) in the U.S. (43). Kim and colleagues reported significantly more childrearing women with disabilities were current smokers (29.58% vs. 15.13%) compared to childrearing women without disabilities in the U.S (37). Finally, another research study revealed that women with disabilities were significantly more likely to currently smoke (30.5% vs. 14.5%) compared to women without disabilities (38).

##### Alcohol and drug use and sexually transmitted infection

Researchers reported that college students with disabilities had significantly higher prevalence rates of past 30-day alcohol use (69.5% vs. 66.6%, respectively), marijuana use (22.2% vs. 15%, respectively), and other drug use (11.6% vs. 4.6%, respectively) compared to those without disabilities in the U.S. (43). Additionally, researchers reported that a significantly higher proportion of women with IDD (6.3%) and multiple disabilities (3.4%) had substance use disorder compared to those without disabilities (0.9%) (45). Researchers in Canada reported that both men (7.1%) and women (11.5%) with disabilities were significantly more likely to report having been diagnosed with a sexually transmitted infection compared to men (5.5%) and women (7.1%) without disabilities (53).

##### Lack of health screening

Lack of health screening is also considered a risk factor for adverse health outcomes in persons with disabilities. Researchers in South Korea reported that persons with disabilities had a significantly lower colorectal cancer screening participation rate compared to persons without disabilities (adjusted odds ratio [aOR] = 0.71) (54). Additionally, same study reported that individuals with specific conditions such as autism (aOR = 0.47), renal failure (aOR = 0.50), brain injury (aOR = 0.58), ostomy (aOR = 0.60), and ID (aOR = 0.61) had the lowest colorectal cancer screening participation rate (54). Furthermore, researchers reported that compared to women without chronic conditions (76.1%), a significantly lower proportion of women with chronic conditions (68.3%) received a PAP test within three years in France (55).

### Social determinants of health and immunization

Under this theme, we presented 12 domains of social determinants of health that we identified in our review. They are immunization, sexual knowledge, poverty, access to clean water and air, violence, functional limitations, education, employment, childhood adversity, gender, race/ethnicity, and attitudes. Accordingly, research revealed that the level of immunization was relatively lower in persons with disabilities (74.63%) compared to persons without disabilities (88.14%) in Sierra Leone (11).

#### Poverty

Researchers revealed that more adults with disabilities were living in poverty or near poverty (48.7%) compared to adults without disabilities (28%) (48). Kim and colleagues reported significantly more childrearing women with disabilities were living in the poverty level (50.47% vs. 38.41%) compared to childrearing women without disabilities in the U.S (37). Besides, researchers found that women with IDD had higher rates of poverty (41.3% vs. 22.1%) compared to women without disabilities in Canada (39). In women with disabilities, researchers found that women with IDD were more likely to live in low-income areas compared to women without IDD (45).

#### Access to clean water and air

Individuals with disabilities (53.73%) had significantly lower levels of access to managed water supply (e.g., pipe) compared to persons without disabilities (58.46%) in Sierra Leone (11). One research also revealed that children with ID were at higher rates of being exposed to harmful chemicals in the air: 33% more likely for diesel particulate matter, 30% for carbon monoxide, and 17% for sulfur dioxide in the UK (56).

#### Violence and functional limitations

Persons with ID were 9.6 to 125 times more likely to experience poor day-to-day activity limitations compared to those without disabilities in Scotland (57).

Researchers reported that (a) men with disabilities (17.7%) were significantly more likely than men without disabilities (13.2%) to be robbed, (b) significantly more women with disabilities (9.5%) experienced sexual violence compared with women without disabilities (6.3%), and (c) men (36.2%) and women (35.5%) with disabilities were both significantly more likely than men (26.1%) and women (27.6%) without disabilities to have been victimized by someone who was known (e.g., neighbor, colleague) (51).

#### Education and employment

Research revealed that a lower percentage of persons with disabilities (24.7%) completed college or above degrees compared to persons without disabilities (43.2%) in the U.S (51). Mitra and colleagues found that more women without disabilities (40.1%) were at least college degree graduates compared to women with disabilities (28.4%) in the U.S. (38).

Researchers revealed that more adults with disabilities were unemployed (7.3%) compared to adults without disabilities (4.5%) in the U.S (48). In South Africa, researchers found that more persons without disabilities (48.40%) were employed compared to persons with disabilities (32.22%) (22). Another research revealed that fewer adults with disabilities and adverse childhood experience exposure (30.2%) were employed compared to their counterparts (65.1%) (58). In women with disabilities, one research documented that childrearing women without disabilities (60.02%) were significantly more likely to be employed compared to childrearing women with disabilities (47.86%) (37).

#### Childhood adversity

Researchers in the U.S. reported that significantly more children with DD (54%) experienced at least one adverse family experience compared to children without DD (45.5%) (59). They also reported that children with DD had a significantly higher prevalence of income insufficiency (30.3% vs. 22.8%), household mental illness (10.8% vs. 7.3%), and household substance use (13.4% vs. 9.9%) (59). Similarly, another research conducted in the U.S. found a higher percentage of persons with disabilities (36.5%) reported high adverse childhood experiences compared to persons without disabilities (19.6%) (58). More specifically, more reported adverse childhood experience includes physical abuse (57.2% vs. 47.5%), verbal abuse (77.9% vs. 70%), being touched sexually by an adult (38.9% vs. 25%), being forced to touch an adult sexually (32.1% vs. 19.1%), and being forced to have sex with an adult (23.7% vs. 13.2%) (58). Consequently, among those with high adverse childhood exposure, persons with disabilities were at a higher risk of being a current smoker (38.2% vs. 30.7%), being obese (44.1% vs. 30.8%), reporting no exercise in the past 30 days (45.3% vs. 18.7%), and reporting HIV risk behaviors (9.7% vs. 9%) compared to persons without disabilities.

#### Gender

Compared to men with disabilities, women with disabilities may experience poorer health outcomes. Researchers in China found that significantly more women with disabilities (64.42%) had high blood lipids compared to men with disabilities (53.37%) (60). Conversely, significantly more men with disabilities had high blood pressure (44.35% vs. 40.89), high blood glucose (21.50% vs. 17.79%), and overweight (51.19% vs. 46.97%) compared to women with disabilities. Another study examined disparities in substance abuse treatment utilization in women with ID and reported that women with intellectual disability/substance abuse (57%) were more likely than men (53%) to have a serious mental illness diagnosis (61). Additionally, research revealed that women with disabilities had higher rates of depression (72.5% vs. 58.4%), anxiety (84.4% vs. 72.1%), severe psychological distress (57.5% vs. 37.2%), and comorbid anxiety and depression (66.4% vs. 52.3%) compared to men with disabilities (51). Finally, researchers reported that women with ID were more likely to report ‘bad’ or ‘very bad’ general health (14.72% vs. 12.51%), have a physical disability (36.52% vs. 29.26%), experience deafness or hearing impairment (10.55% vs. 6.97%), and have blindness or a visual impairment (13.14% vs. 9.98%) than men with ID (57). The same research revealed that men with ID (18.72%) were more likely to report a mental health condition compared to women with ID (16.29%) (57).

#### Race/ethnicity

Researchers examined racial/ethnic disparities in persons with disabilities. One study revealed that a significantly higher percentage of Black community-dwelling persons aged 65 and older had obesity (25% vs. 11%), arthritis (59% vs. 54%), diabetes (23% vs. 14%), and functional limitations (20% vs. 15%) compared to their White counterparts in the U.S (62). Conversely, a significantly higher percentage of White community-dwelling persons aged 65 and older had hip fractures (5% vs. 2%), other fractures (5% vs. 2%), and heart attacks (17% vs. 14%) compared to Black counterparts (62). Another longitudinal study conducted in the U.S. revealed that (a) Black and Hispanic women had the highest disability levels (i.e., levels of functional limitations), (b) White women and racial/ethnic minority men had intermediate disability levels, and (c) White men had the lowest disability levels at baseline (63).

Researchers also found American Indians-Alaskan Natives had a higher risk for developing disability compared to Hispanic White people, non-Hispanic Asians, and Hispanics in the U.S. (64). Finally, researchers revealed that Black and Latino persons with IDD in the U.S. had less income and education, were more likely to be uninsured, and lived in urban areas compared to White persons with IDD (65). They also reported that Latino persons with IDD were 1.72 times more likely than White persons with IDD to be obese (65).

#### Attitudes

Persons with disabilities experience negative attitudes from society. Researchers found that individuals with disabilities were more than three times more likely to be exposed to discrimination compared to those without disabilities in the UK (66).

This discrimination manifests in various forms, including barriers to employment, inequities in healthcare access, and social exclusion. The impact of such discriminatory practices extends beyond immediate social interactions, contributing to long-term adverse effects on mental and physical health. These findings highlight the urgent need for comprehensive policies and societal changes aimed at promoting inclusivity and equality. Efforts must include raising public awareness, enforcing anti-discrimination laws, and ensuring equal opportunities in all aspects of life for persons with disabilities. Addressing these issues is not only a matter of social justice but also essential for the overall well-being and integration of individuals with disabilities into society.

## Discussion

This scoping review aimed to provide a comprehensive overview of health inequities experienced by 1.3 billion individuals with disabilities globally. By searching and rigorously screening quantitative literature from 2011 to 2022 across several databases, supplemented by manual searches of reference lists, we identified 51 scholarly articles on health inequities among people with disabilities. The synthesis of evidence from our scoping review elucidates the pervasive health inequities experienced by individuals with disabilities globally compared to their counterparts without disabilities across various domains (i.e., access to healthcare and resources, morbidity, mortality, and risk factors, social determinants of health).

Access to quality healthcare services and resources remains a fundamental determinant of health outcomes (2022). However, our review underscores the persistent challenges and disparities encountered by individuals with disabilities in accessing healthcare (10), exacerbating existing health inequities (3). The findings resonate with previous research highlighting the heightened odds of unmet medical needs, inadequate dental care, and medication non-adherence among individuals with disabilities (3, 8). Notably, the barriers to healthcare access are multifaceted, encompassing financial constraints, transportation limitations, and structural inadequacies within healthcare systems (23, 25, 67). Addressing these barriers necessitates a holistic approach, involving policy reforms, infrastructure enhancements, and provider training initiatives to ensure equitable access to healthcare for individuals with disabilities (27–34).

Individuals with disabilities are disproportionately burdened by comorbid physical health conditions, compounding their health challenges and exacerbating disparities in health outcomes (35, 43). Adults with disabilities are more likely to die from heart disease, cancer, and stroke (68). The synthesis of quantitative evidence reveals the elevated prevalence rates of various chronic conditions, including diabetes, asthma, cardiovascular diseases, and musculoskeletal disorders, among individuals with disabilities across different age groups and geographical contexts (8). These findings underscore the critical need for targeted interventions aimed at addressing the complex interplay between disability and other physical health concerns (37). Health promotion efforts should prioritize preventive strategies, early detection, and comprehensive management of comorbidities to mitigate the adverse health outcomes experienced by individuals with disabilities.

In addition to physical health disparities, individuals with disabilities face heightened risks of comorbid mental health conditions, further elucidating the intricate relationship between disability and mental well-being (36). Our review documents elevated prevalence rates of mood disorders and anxiety disorders among individuals with disabilities across diverse populations (36). These findings highlight the imperative for integrated approaches to healthcare service delivery that prioritize mental health screening, early intervention, and access to culturally responsive mental health services for individuals with disabilities (36). This suggests that destigmatizing mental illness and promoting mental health literacy within disability communities should be emphasized in order to foster resilience and facilitate help-seeking behaviors.

The disparities in health outcomes are starkly reflected in the elevated mortality rates observed among individuals with disabilities, accentuating the urgent need for targeted interventions to address premature deaths within this population (5). Our review unveils compelling evidence documenting the heightened risks of mortality across different age groups and health conditions among individuals with disabilities (42). From childhood to older adulthood, individuals with disabilities face significantly higher odds of premature mortality, necessitating a comprehensive public health response. Mitigating mortality disparities demands a multifaceted approach encompassing early intervention, preventive healthcare services, and holistic care coordination to address the complex health needs of individuals with disabilities throughout their lifespan (3, 48).

The identified risk factors shed light on the multifaceted determinants influencing health outcomes among individuals with disabilities, encompassing lifestyle behaviors, psychosocial factors, and structural issues. With physical inactivity, poor sleep hygiene, substance use, and lack of health screening, to name a few, individuals with disabilities are disproportionately affected by a myriad of risk factors that contribute to adverse health outcomes (3, 38, 48, 55, 57). Addressing these risk factors necessitates tailored interventions that prioritize health promotion (1), behavior change strategies, and structural reforms aimed at creating supportive environments for individuals with disabilities to thrive (1).

The social determinants of health play a pivotal role in shaping health outcomes and disparities among individuals with disabilities, reflecting broader structural inequities and societal attitudes toward disability (1). Our review elucidates the complex interplay between social determinants, disability, and health outcomes across diverse contexts. From poverty and violence to education and employment, individuals with disabilities face intersecting barriers that perpetuate health inequities and exacerbate disparities (47). Addressing social determinants demands a holistic approach that addresses systemic barriers, promotes inclusive policies, and fosters community empowerment to create environments that support the health and well-being of individuals with disabilities.

Although our scoping review depicted a comprehensive literature landscape of quantitative research on the persistent health inequality experienced by people with disabilities globally, it is not without limitations. First, the inclusion criteria of this review were limited to peer-reviewed journal articles published in English and indexed in CINAHL, MEDLINE, and PsycINFO. While these criteria were necessary to ensure methodological rigor and consistency, they introduced a publication bias that likely excluded valuable research from non-English-speaking countries and underrepresented regions. This limitation restricts the global representation of health inequities and may disproportionately omit studies from low- and middle-income countries, where health disparities may be most pronounced. Second, the search terms were designed to focus on core concepts of health inequities and disability, but a more comprehensive list of keywords or inclusion of additional databases may have yielded a broader representation of literature. Future studies should consider a more extensive search strategy to capture a wider range of studies, including those from grey literature and diverse geographic regions. Third, a significant limitation of this review is the exclusion of 127 manuscripts due to our inability to retrieve their full texts. These missing manuscripts could have contributed valuable insights to the review, and their absence highlights the challenges of accessing research, particularly in resource-constrained settings. We recommend future research efforts prioritize improved accessibility to primary sources and collaboration with global partners to ensure comprehensive coverage. Finally, this scoping review focused solely on quantitative studies. This decision was made because quantitative data was more aligned with our study objectives, which sought to identify measurable outcomes such as prevalence rates in different conditions. However, we recognize the value of qualitative studies and suggest their inclusion in future research to provide a more comprehensive understanding of the topic.

## Conclusion

In conclusion, our scoping review offers a comprehensive examination of global health inequities for individuals with disabilities, highlighting the multifaceted challenges and disparities that persist across various domains. The synthesized evidence underscores the urgent need for concerted efforts to address the structural, social, and individual determinants contributing to health inequities experienced by disability populations.

Targeted interventions, policy reforms, and research initiatives are imperative to promote health equity, foster inclusive healthcare systems, and advance the well-being of individuals with disabilities globally. Development of innovative career pathways for health professionals with interest in working with persons with disabilities must be considered and supported by universities and institutions for higher learning. In the U.K., a call to action was made to include nursing courses that address the needs of disabled persons and consider such specialty (69). Preparation of health care workforce to provide health services with persons with disabilities is essential for access to care and promoting safe and competent case (68). By prioritizing disability-inclusive approaches and amplifying the voices of disability communities, we can collectively strive toward a more equitable and inclusive future where all individuals, regardless of ability, have the opportunity to attain the highest standard of health and well-being.

## Data Availability

The original contributions presented in the study are included in the article/supplementary material, further inquiries can be directed to the corresponding author.
